# Comparing Methods for Missing Paternal Linkages in Administrative Data

**DOI:** 10.23889/ijpds.v9i5.2791

**Published:** 2024-09-18

**Authors:** Amani Hamad, Barret Monchka, Oleguer Plana-Ripoll, Olawale Ayilara, Lisa Lix

**Affiliations:** 1 University of Manitoba; 2 Aarhus University

## Objective

Administrative data often lacks complete family linkages, particularly to fathers, hindering familial health research. We compared methods to address missing paternal linkages in research investigating the familial transfer of mental disorders.

## Approach

A population-based cohort study of Manitoba (Canada) born adults, +18 years old between 1977 and 2017 with maternal linkages. Three methods were used to address missing paternal linkages: indicator category, complete case, and multiple imputation, which identified three candidate fathers for each individual within -4 to +7 years of mother’s age with same postal code. For each method, the association of maternal and paternal history with mental disorder risk during follow-up was tested using multivariable logistic regression models adjusted for demographics and comorbidities.

## Results

The cohort included 142,549 individuals; 22.6% lacked paternal linkages. Using indicator category, maternal and paternal histories were associated with mental disorder risk, with odds ratio (OR) 1.49, 95% confidence interval (CI): 1.45-1.52 and OR 1.34, 95% CI: 1.30-1.37, respectively. Similar results were obtained in the complete case analysis (OR 1.49, 95% CI: 1.45-1.53 and OR 1.33, 95% CI: 1.30-1.37, for maternal and paternal history, respectively). With multiple imputation, maternal history’s association with mental disorder risk remained consistent with the other methods (pooled OR 1.52, 95% CI: 1.49-1.56); paternal history was associated with a smaller risk (pooled OR 1.25 95% CI: 1.22-1.29).

## Conclusions and Implications

The three methods for addressing missing paternal linkages produced similar findings about the familial transfer of mental disorders. Future familial studies could incorporate multiple methods to demonstrate robustness of findings.

